# Dosage form suitability in vulnerable populations: A focus on paracetamol acceptability from infants to centenarians

**DOI:** 10.1371/journal.pone.0221261

**Published:** 2019-08-20

**Authors:** Fabrice Ruiz, Thibault Vallet, Amélie Dufaÿ Wojcicki, Émilie Belissa, Jean-Eudes Fontan, Loïc de Pontual, Sylvie Nathanson, Alain Chevallier, Sandra Laribe-Caget, Vincent Boudy

**Affiliations:** 1 ClinSearch, Malakoff, France; 2 Département innovation pharmaceutique, Agence Générale des Équipements et Produits de Santé (AGEPS), Assistance Publique des Hôpitaux de Paris (AP-HP), Paris, France; 3 Hôpital Jean Verdier, Groupe Hospitalier Universitaire Paris Seine-Saint-Denis, Assistance Publique des Hôpitaux de Paris (AP-HP), Bondy, France; 4 Centre Hospitalier de Versailles - André Mignot, Le Chesnay, France; 5 Hôpital Broca, Groupe Hospitalier Universitaire Paris Centre, Assistance Publique des Hôpitaux de Paris (AP-HP), Paris, France; 6 Hôpital Rothschild, Groupe Hospitalier Universitaire Est Parisien, Assistance Publique des Hôpitaux de Paris (AP-HP), Paris, France; 7 Université Paris Descartes, Paris, France; University of Tennessee Health Science Center, UNITED STATES

## Abstract

**Introduction:**

Medicine acceptability is a multi-faceted concept driven by both product and user characteristics. Although a key factor for treatment effectiveness, especially in vulnerable populations, knowledge of those medicine features that best promote individual user acceptability remains fragmented. Focusing on paracetamol, this study has explored the appropriateness of pharmaceutical products in different dosage forms to achieve adequate patient acceptability from infants to centenarians.

**Methods:**

This observational, multicentre, prospective study was carried out in 10 hospitals, 8 nursing homes and over 150 community dispensaries. Observers reported several behaviours/events evaluating acceptability for 1016 different pharmaceutical product uses in paediatrics (<18y.) and 1288 in the elderly (≥65y.). Using mapping and clustering, a multivariate approach offered an intelligible reference framework for each population, providing comprehensive scores: positively or negatively accepted.

**Results:**

Among all the evaluations supporting the acceptability reference frameworks, there were 502 reports on paracetamol products intake. Herein we focused on the 5 products with ≥30 evaluations. Although oral suspension and powder for oral solution were positively-accepted in the paediatric group, the powder had a higher rate of negative patient reaction (p<0.001). Of those that received this formulation, 72% were ≤8y., and therefore suitable to receive the better accepted oral suspension. In the elderly, patients with swallowing disorders were preferentially treated with such powders (p<0.001), which were less often fully taken than orally disintegrating tablets (p<0.001). Even in those patients ≥90y., capsule formulations appeared to be the best accepted product in patients without swallowing alterations, and thus could be a suitable alternative to the powder in this population.

**Conclusions:**

By better integrating patient characteristics when choosing dosage forms, clinicians and caregivers may improve treatment acceptability and adherence. Moreover, hospitals and healthcare institutions could optimise purchasing to best suit their local population, disseminating information to help staff align specific dosage forms to targeted patients.

## Introduction

How many healthcare professionals consider all the characteristics of their patients prior to prescribing or delivering a specific preparation of paracetamol (acetaminophen)? Beyond contraindications, such as avoiding sodium loaded effervescent tablets in patients at risk of hypertension [1], or specific excipients due to intolerances, dozens of alternatives are available on the market to select the medicinal product best adapted to obtain an adequate acceptability for the concerned patient.

In the context of medicinal product administration, the earliest occurrence of the word “acceptability” that we have found in Pubmed was published in 1965 [2]. Since, the number of manuscripts addressing this concept has steadily increased. Acceptability of medicinal products has emerged as a major factor in compliance, and consequently as a key element for treatment effectiveness, especially in vulnerable populations [3].

In their role as a regulatory body, the European Medicines Agency (EMA) included a full section dedicated to acceptability in their 2013 guideline on the pharmaceutical development of medicines for paediatric use [4]. Therein, applicants were encouraged to integrate the evaluation of patient acceptability as a vital part of pharmaceutical and clinical development. The EMA has defined patient acceptability as, “The overall ability and willingness of the patient to use and its caregiver to administer the medicine as intended” [4]. This implies an interrelationship between users (patients and caregivers) and their characteristics (age, frailty, culture…) on one side, and on the other, the medicine and its characteristics (excipients, size, texture, dose, device…). The combination of all of these characteristics must coalesce to result in proper preparation and administration, and in general a good acceptability of the medicinal product for the user.

The importance of acceptability of medicines is not limited to paediatrics, as highlighted by the 2009 ICH Q8 guideline: “in all cases, the product should be designed to meet patients’ needs and the intended product performance” [5]. Adequate acceptability is thus crucial for all vulnerable patients, including those of the older population (≥ 65 years of age). The EMA called attention to this issue in 2017 through the draft version of their reflection paper on the pharmaceutical development of medicines for use in the older population [6]. Manufacturers and clinicians have thus been encouraged to consider the typology of each user individually to reach an adequate acceptability by proposing well suited treatments.

Acceptability is a multidimensional concept simultaneously driven by a composite of factors belonging to both the user and the medicinal product. Despite the numerous tools used to assess acceptability, a recent review was unable to identify a standardized methodology [7]. Furthermore, none of the referenced studies simultaneously considered the different dimensions of acceptability as defined by the EMA guideline [4]. Statistical processing of acceptability has generally been restricted to univariate data analysis, even in those cases where several measures were collected and a multivariate approach would have been more appropriate [8].

In this study, we have adopted such a multivariate approach, concurrently exploring many different facets of acceptability. Focusing on paracetamol, the active principal ingredient (API) most frequently used in France [9], which is also widely used in Europe and throughout the world [10, 11], we have observed and analysed the acceptability of many medicinal products with various dosage forms, in patients ranging from infants to centenarians.

## Methods

**Fig 1:**
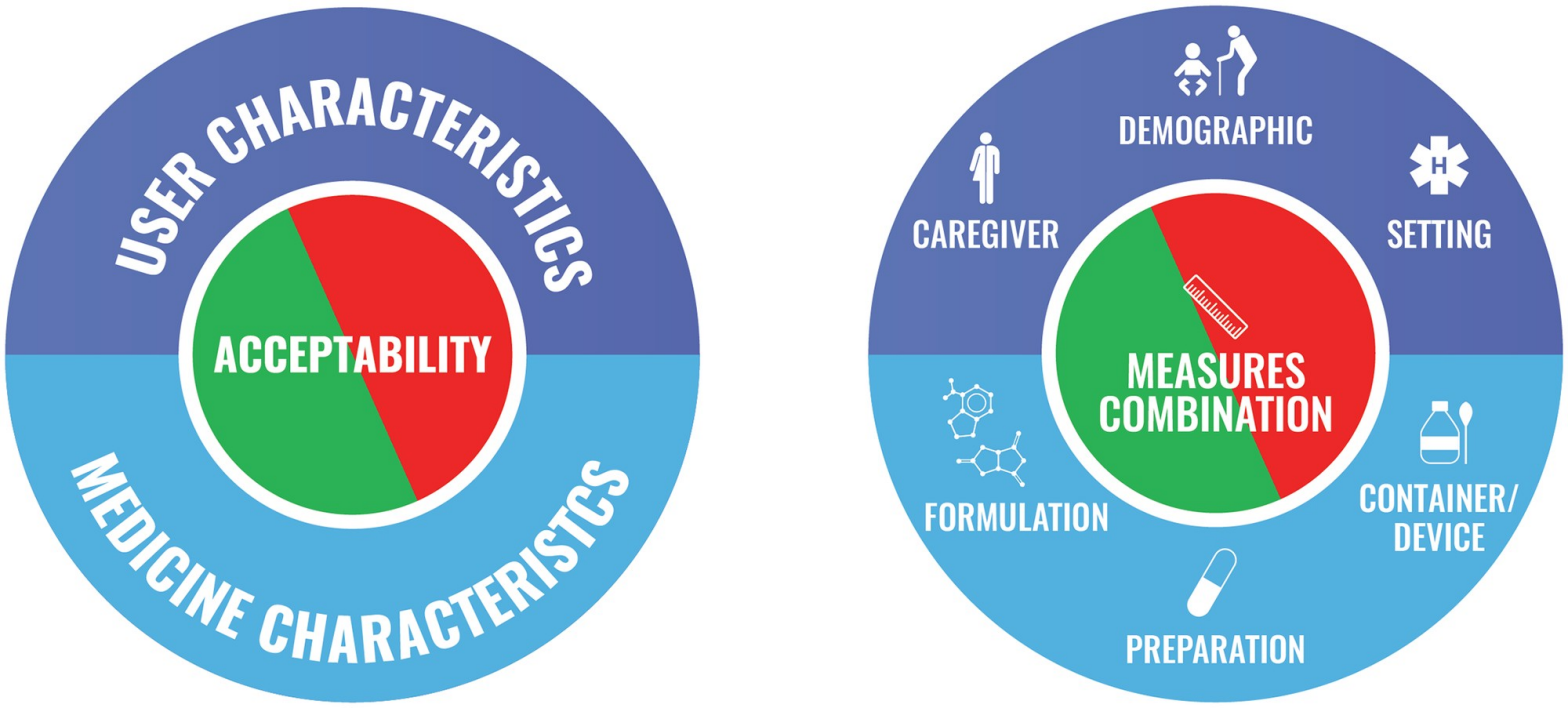
Acceptability: A multidimensional concept.

To assess the multi-faceted concept of acceptability (Fig 1), we used a novel multivariate approach: CAST—ClinSearch Acceptability Score Test [12–14].

Based on real-life, observed data, the questionnaire examines the following observable contents:

- results of intake (the required dose fully, partly or not taken at all),
- patient reaction during the administration (positive, neutral or negative reaction),
- time needed to prepare (from opening any packaging to having a required dose of medication ready to use, including all handling and modifications),
- time needed to administer the required dose of medication (from a required dose of medication ready to use to the end of the intake),
- recourse to methods to ease/achieve administration (dividing the intake of a dose which cannot be taken as a whole, altering the intended use [modify dosage form such as tablet crushed or capsule opened; use a device not provided; use another route/mode of administration], use of food/drink to mask a taste or ease swallowing, patient reward, use of restraint).

Based on data distribution and the clinical practice expertise of the authors, the preparation and administration time (10 second accuracy) was classified as short, medium or long. These scores were defined as less than 20 seconds, from 21 seconds to 1 minute, or longer than 1 minute, in the older inpatient population [14], and as less than 1 minute, 1 to 2.5 minutes, or longer than 2.5 minutes, in paediatric patients [12].

Each evaluation of one medicinal product, taken by one patient, corresponded to a particular combination of observed measures.

A multiple correspondence analysis (MCA) summarised the key information contained in the data on a 3-dimensional acceptability map, then a hierarchical clustering process on principal components (HCPC) gathered the evaluations into clusters. The “positively accepted” evaluations naturally emerged in a different cluster than those which were “negatively accepted”, defining two different acceptability profiles. This acceptability reference framework proposed in a targeted population allows for standardised scoring for medicinal products, and the assessment of both user and medicine characteristics impact on acceptability scores.

This observational study used two acceptability reference frameworks, one for older patients and a second for the paediatric population. The data in this study were exempt from review by an institutional review board as confirmed by one ethics committee (Comité de Protection des Personnes Ile de France VII). Approvals were obtained from the Advisory Committee for Data Processing in Health Research (CCTIRS—"Comité Consultatif sur le Traitement de l’Information en matière de Recherche dans le domaine de la Santé") and the French Data Protection Authority (CNIL—"Commission Nationale de l’Informatique et des Libertés"). Freely given informed consent has been obtained from every subject (or legal representative) prior participation in the study.

These frameworks were based on large sets of medicinal product use evaluations collected between May 2015 and August 2018. Patients were recruited at random, on a voluntary basis, by many doctors and pharmacists from ten hospitals, eight nursing homes, and more than 150 community dispensaries throughout France. Age was the only eligibility criterion. We also excluded any infusions administered when a catheter was already in place, considering that the insertion of the catheter–which belongs to the administration sequence of the medicine–would be lacking from the data collected. Following study inclusion, the caregiver for outpatients, or the healthcare professional for inpatients, completed the questionnaire related to the observed use of the medicinal product. In cases of polymedication, only the first treatment administered was observed.

To explore the factors influencing acceptability we focused in this study on paracetamol medicinal products.

When a specific medicinal product was evaluated by at least 30 patients in the set of evaluations, the barycentre of these evaluations was positioned on the acceptability map. The acceptability score was structured by the acceptability profile of their barycentre and the proportion of confidence ellipses belonging to it, as previously described [12–14]. Similarly, the acceptability of these medicinal products in subpopulations of patients were studied.

Statistical tests were used to assess the significance of the differences observed between the variables for each distinct paracetamol medicinal product. Pearson’s Chi-squared test was used when there was a minimum expectation of 5 for 80% of cells without any null expectation [15]. When there were fewer patients observations, Fisher’s exact test provided an alternative to surmount this difficulty.

R and SAS were used. The R packages FactoMineR [16] and MissMDA [17] were used to perform MCA and HCPC, and to handle missing data. Data analyses in R and results were checked with SAS 9.4 for patients with complete data.

## Results

### Populations and paracetamol medicinal products

A total of 2304 evaluations describing the use of many different medicinal products support the acceptability reference framework that was built for each population (1016 in paediatrics and 1288 in the elderly). Among these evaluations, there were 502 observer reports on the intake by an individual patient of one paracetamol product. Among the 37 distinct paracetamol medicinal products assessed, five had ≥30 evaluations.

#### Paediatric population

Among the 283 medicinal products assessed in the 1016 evaluations supporting the acceptability reference framework in paediatrics (S1 and S2 Tables), there were 18 distinct paracetamol products. These included eight different concentrations (2.4g/100ml, 3g/100ml, 100mg, 150mg, 200mg, 300mg, 500mg and 1000mg) and nine dosage forms (oral suspension, oral solution, powder for oral solution, suppository, capsule, tablet, coated tablet, effervescent tablet and oral lyophilizate), produced by four different marketing authorisation holders. Table 1 presents those medicinal products that were assessed in more than five patients. All of the products shown below were manufactured by the same marketing authorisation holder.

**Table 1:** Demographic characteristics of the paediatric population patients administered the studied paracetamol medicinal products with greater than five assessments.

Characteristics of patients		2.4% Oral suspension (n = 199)	300mg Powder for oral solution (n = 39)	500mg Powder for oral solution (n = 22)	100mg Suppository (n = 19)	500mg Capsule (n = 14)	500mg Tablet (n = 8)	100mg Powder for oral solution (n = 7)	150mg Suppository (n = 6)
**Gender**	Girls	51%	45%	50%	61%	57%	62%	57%	17%
		*md*: *7*	*md*: *1*		*md*: *1*				
**Age** (years)	[birth; 2]	64%	8%	0%	100%	0%	0%	43%	100%
	[3; 5]	25%	26%	0%	0%	0%	0%	29%	0%
	[6; 8]	11%	38%	32%	0%	0%	12%	29%	0%
	[9; 11]	0%	26%	50%	0%	57%	75%	0%	0%
	[12; 14]	0%	3%	18%	0%	36%	12%	0%	0%
	[15; 17]	0%	0%	0%	0%	7%	0%	0%	0%
		*md*: *6*			*md*: *1*				
**Place**	Hospital	45%	59%	68%	0%	86%	38%	0%	0%
	Ambulatory	55%	41%	32%	100%	14%	62%	100%	100%
		*md*: *2*			*md*: *2*				
**Exposure**	First intake	11%	15%	18%	37%	29%	0%	29%	17%
		*md*: *4*							

md: missing data

#### Older population

Among the 315 medicinal products assessed in the 1288 evaluations supporting the acceptability reference framework in the older inpatient population (S3 and S4 Tables), there were 19 distinct paracetamol products. There were three strengths (10mg/1ml, 500mg and 1000mg), seven formulations (capsule, tablet, coated tablet, effervescent tablet, orally disintegrating tablet (ODT), powder for oral solution and solution for injection), and eight marketing authorisation holders. Table 2 presents those paracetamol products assessed in more than five patients, these three paracetamol products all had the same marketing authorisation holder.

**Table 2:** Demographic characteristics of the older inpatients administered the studied paracetamol medicinal products with greater than five assessments.

Characteristics of patients		500mg Capsule (n = 76)	500mg Orally disintegrating tablet (n = 34)	500mg Powder for oral solution (n = 30)
**Gender**	Women	71%	74%	63%
**Age** (years)	[65; 75]	4%	0%	0%
	[75; 85]	42%	41%	23%
	[85; 95]	39%	53%	53%
	[95; 110]	15%	6%	23%
**Place**	Hospital	82%	100%	100%
	Nursing home	18%	0%	0%
**Exposure**	First intake	3%	79%	3%
		*md*: *4*		*md*: *1*
**Disorders**	Swallowing disorder	4%	18%	30%
		*md*: *2*		
	Memory disorder	59%	24%	70%
		*md*: *2*		
	Muscular or rheumatologic disorders of the upper limbs	35%	15%	43%
		*md*: *2*		

md: missing data

### Paracetamol medicinal products acceptability scores

**Fig 2:**
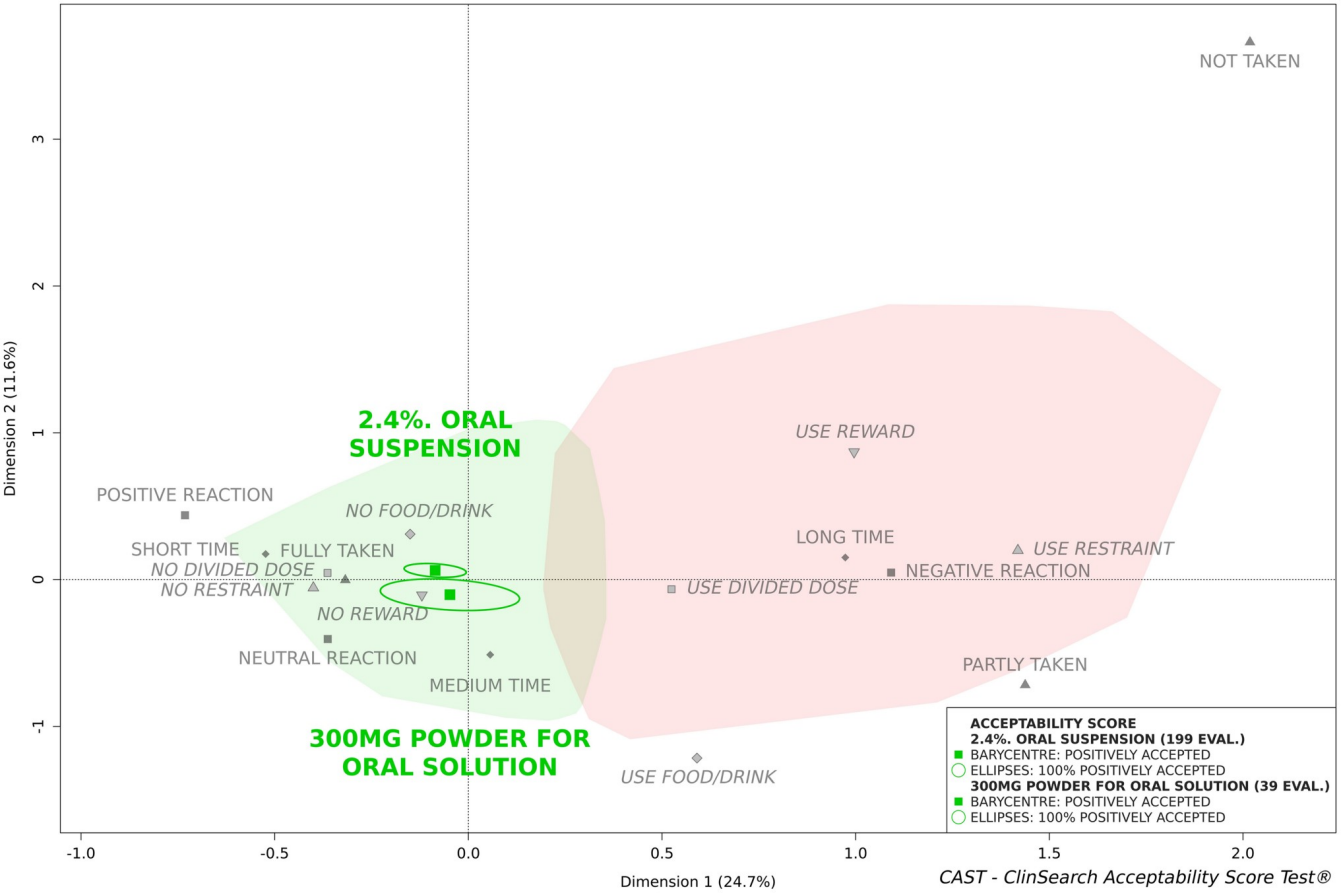
Oral suspension (2.4% paracetamol) and Powder for oral solution (300mg paracetamol) acceptability scores in the paediatric population.

Fig 2 presents the acceptability scores of the two paracetamol medicinal products that were assessed more than 30 times in the paediatric population: 2.4% oral suspension and 300mg powder for oral solution.

The greatest number of evaluations was obtained for the 2.4% oral suspension, 77% were gathered into the “positively accepted” profile zone compared to 74% for the 300mg powder for oral solution. The barycentres of their evaluations have been assigned to the "positively accepted” profile as well as 100% of the confidence ellipses.

**Fig 3:**
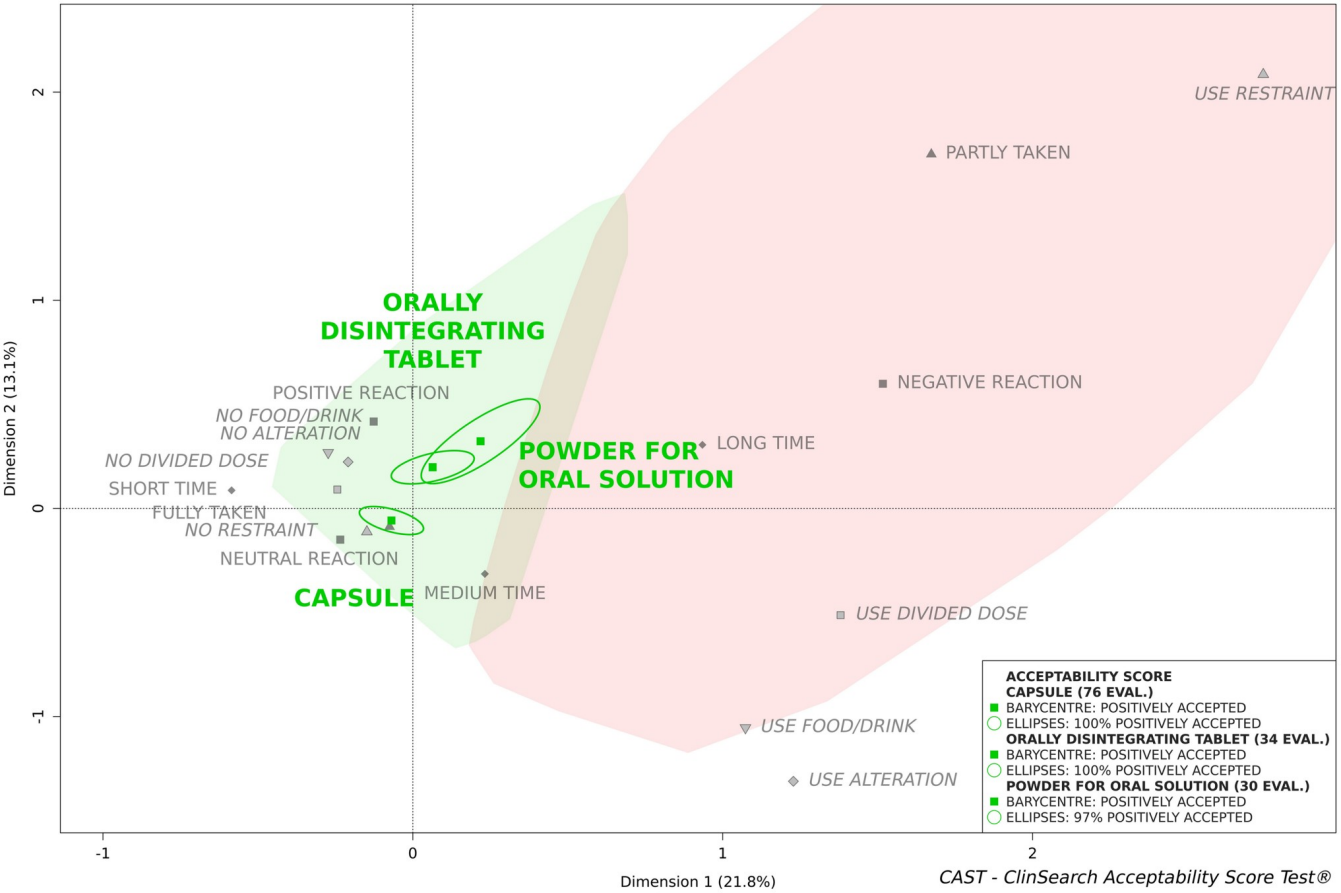
500mg paracetamol acceptability profile in geriatric population, comparison of 3 dosage forms: Capsule, orally disintegrating tablet and powder for oral solution.

Fig 3 presents the acceptability scores of the three paracetamol medicinal products, in the elderly population, that were assessed more than 30 times. These were 500mg capsules, 500mg orally disintegrating tablets and the 500mg powder for oral solution.

The greatest number of evaluations was obtained for the capsule, 89% were gathered into the “positively accepted” profile zone compared to 88% for the ODT and 73% for the powder for oral solution. The barycentres of all of their evaluations have been assigned to the "positively accepted” profile, together with 100% of their confidence ellipses, with the exception of a small part of the ellipse, in the third dimension, for the powder for oral solution.

The paracetamol medicinal products assessed in this study appeared to be accepted in both of the populations studied, as the barycentre, along with the entire confidence ellipses, with the exception of the powder for oral solution in the elderly, belonged to the "positively accepted" profile. These dosage forms seem appropriate for the patients of these two populations.

Objective comparisons between these different “positively accepted” products are possible; their barycentre positions on the map permit ranking. In this study, although the powder for oral solution was classified as positively accepted by the paediatric and almost similar in the older populations, it was not the best accepted dosage format in either. Its barycentre fell into the furthest limit of the zone of positively connoted categories on the map. For the older inpatients, ODT tended to be more positively accepted than the powder for oral solution, but ODT presented a lower acceptability profile than capsules in this population. These results reflect differences in the observational measures.

Other formulations have not been presented on the map, but even in these cases with fewer than 30 evaluations some tendencies could be observed. The powder for oral solution with a higher strength (500mg), taken by older children, was positioned closer on the map to positively connoted categories, while a lower strength (100mg), taken by younger children, was positioned more distally from them. For those patients under 3, even though the 100mg and 150mg suppository had a better score than the 100mg powder for oral solution, the 2.4% oral suspension of paracetamol was located closest to the ideal position on the map.

### Constituting variables

Each evaluation reflects the relation of a specific treatment with a specific user. The barycentre of evaluations summarises in one point the variability of uses; it takes into consideration combinations of evaluations which themselves are composed of combinations of measures.

Table 3 presents the distribution, per treatment, of the related observational measures.

**Table 3:** Observational measures per variables.

	Paediatric population		Older population		
	2.4% oral suspension (n = 199)	300mg powder for oral solution (n = 39)	500mg capsule (n = 76)	500mg orally disintegrating tablet (n = 34)	500mg powder for oral solution (n = 30)
**Result of the intake**					
Fully taken	176 (88)	31 (82)	75 (100)	34 (100)	24 (80)
Partly taken	21 (11)	7 (18)	0 (0)	0 (0)	6 (20)
Not taken	2 (1)	0 (0)	0 (0)	0 (0)	0 (0)
		*md*: *1*	*md*: *1*		
**Patient’s reaction**					
Positive reaction	105 (53)	5 (14)	6 (8)	4 (12)	1 (3)
Neutral reaction	49 (25)	15 (40)	65 (87)	23 (70)	23 (77)
Negative reaction	45 (22)	17 (46)	4 (5)	6 (18)	6 (20)
		*md*: *2*	*md*: *1*	*md*: *1*	
**Prep.-admin. Time**					
Short time	116 (59)	22 (58)	33 (43)	2 (6)	3 (10)
Medium time	48 (24)	10 (26)	34 (45)	2 (6)	13 (43)
Long time	33 (17)	6 (16)	9 (12)	30 (88)	14 (47)
	*md*: *2*	*md*: *1*			
**Divided dose**	140 (70)	12 (31)	27 (36)	2 (6)	6 (20)
**Alteration**	2 (1)	0 (0)	5 (7)	1 (3)	0 (0)
**Food/drink**	21 (11)	7 (18)	8 (11)	2 (6)	1 (3)
**Reward**	14 (7)	0 (0)	**⧫**	**⧫**	**⧫**
**Restraint**	39 (20)	7 (18)	1 (1)	1 (3)	5 (17)

number and percentages: n(%)—md: missing data -**⧫** Data not collected in older population

As such, observations must take into account those differences in each population, as shown in Tables 1 and 2. Children administered the 2.4% oral suspension dosage form were significantly younger than those administered the powder for oral solution (p<0.001). In the oldest population, those patients that were administered each of the three paracetamol medicinal products differed for several characteristics. Patients suffering from swallowing disorders were underrepresented in the capsule group and preferentially treated with the powder for oral solution (p<0.001). Moreover, in nursing homes capsules were the sole dosage form observed on five or more occasions. Those patients treated with ODT had fewer cases of muscular (p = 0.03) or memory disorders (p<0.001) than patients treated with the other two dosage forms, and they were more often naïve to previous exposure (p<0.001). This last finding is likely due to the recent introduction of this dosage form at one of the recruiting sites.

Focusing on observations of medicinal product use, the powder for oral solution presented a lower rate of dose fully taken and a higher rate of negative reactions in both populations. The capsule and the ODT were the two formulations that were systematically fully taken in the elderly, while the powder for oral solution was less often fully taken (p<0.001). For more than 50% of the evaluations of the oral suspension use, observers reported a positive reaction of the patient. This oral suspension is flavoured with strawberry and used principally in children under 6 years old, 89% of the paediatric patients were treated with this medicinal product.

In the older inpatient population, preparation and administration time were significantly different for each medicinal product. This was highlighted by the median times observed: 30 seconds for the capsule, 55 seconds for the powder for oral solution and 115 seconds for the ODT.

Paradoxically the prescribed dose of the most accepted dosage form in both populations was divided significantly more often than other forms (p<0.001 in the paediatric population; p = 0.003 in the older population). In the youngest population, this was notably observed in hospital: a single bottle of oral suspension was used for several patients, but the syringe was systematically changed. Among the questionnaire choices for methods used to ease/achieve administration, nurses thus entered that the syringe provided by the manufacturer had not been used. However, during the data review it was decided not to consider this as an alteration of intended use: the procedure had clearly been established by hospital staff for hygienic reasons. Moreover, this will facilitate international comparisons, as in many countries syringes are not provided with oral suspensions. By contrast, in cases where parents reported the use at home of a syringe provided with another medicinal product, this has been coded as an alteration of the intended use.

In the older inpatient population, to avoid an interpretation bias related to differences in the strength prescribed, we verified the absence of significant differences in each group. The ratio of patients with a prescription for 2 or more units (capsule, sachet or tablets) was high for all three groups: 89% with capsule (md: 15), 77% with ODT (md: 8) and 86% with powder for oral solution (md: 2) (p = 0.34). Interpretation of the observed number of doses divided must therefore take into account that the majority of the patients were obliged to take 2 unit doses for each intake.

Alteration of the medicinal product prior to administration was only reported in the older inpatient population. For two patients treated in a nursing home the contents of their capsules were mixed once with coffee served with milk, and in the second case with applesauce. For another patient, treated in a hospital, the content of his capsule was mixed in water. The fourth report of alteration concerned a patient that kept the capsule in their mouth waiting for its dissolution. For one patient the use of another route/mode of administration was reported, indeed the ODT was swallowed. No swallowing disorders were diagnosed for any of these patients.

Food or drink taken just before or after the administration to mask the taste or ease swallowing, were used more often in the paediatric population for the powder for oral solution, but not among older inpatients. Whereas, restraint was significantly over represented with the powder for oral solution in our dataset from the elderly population (p = 0.006), effectively the observers had more often reported that patients had to force themselves to manage to drink the solution.

## Discussion

The acceptability profiles of five medicinal products of a single active principal ingredient, paracetamol, in four different dosage forms were analysed in two populations at each extremity of the life-cycle, including patients from infants to centenarians. Data from further medicinal products were also collected, providing preliminary results for certain subgroups of patients. Underlying conditions appear to have been the principal driver for caregivers and healthcare professionals in choosing the most appropriate dosage form for older patients, while age appears to have most influenced the choices made for the paediatric patients. Of the five complete medicinal product profiles analysed, powder for oral solution was the least well-accepted in both populations. In the older inpatient population, the most accepted formulation identified was the capsule; it was even used in those 11 (14%) patients over 95 years of age that were still able to consume 2 units in a single sitting. Among those 13% of older inpatients that had swallowing disorders, powder for oral solution seems to have been preferentially prescribed (50%). But as swallowing disorder may be screened by the water-swallowing test, it was not surprising that individuals unable to drink a half-glass of water with no interruptions or difficulties were less amenable to the administration of 1000mg of paracetamol dissolved in a half-glass of water. A limited set of data regarding the recently introduced ODT dosage form implied that this form of administration could represent an interesting alternative in terms of acceptability for patients with swallowing disorders. In the paediatric population, paracetamol oral suspension had the best profile of acceptability. Nonetheless, children over five were quite often switched to powder for oral solution even though acceptability was not improved, especially in hospital. This suggests that healthcare professionals and caregivers categorised certain dosage forms to specific ages in the paediatric population. In this case, however, the summary of product characteristics (SmPC) for the oral suspension of paracetamol indicates that it is appropriate for children weighing from 3kg to 26kg (approximately birth to nine years old), thus providing a single, well-accepted liquid dosage form that may be used until the patient might be switched directly to capsules.

Using our multivariate approach, we were able to test the acceptability of different medicinal products of the same API while simultaneously accounting for individual patient characteristics. Multivariate exploratory data analysis provided an in-depth, nuanced understanding of acceptability. Given similar sample sizes, the larger confidence ellipse for the powder for oral solution revealed a greater heterogeneity of patient evaluations compared with other paracetamol dosage forms. This variability implied that subgroups of patients might be identified. In this case, swallowing disorders were present in 30% of the older population receiving the powder for oral solution dosage form, and preliminary results from patients administered an ODT dosage form suggest that it may offer a better accepted alternative. As this was an observational study, however, we were unable to influence the use of any particular dosage form, and at present additional data from dysphagic patients in the older population will be required to adequately test this hypothesis. Similarly, the powder for oral solution was not optimal in the paediatric population; again due to the study’s non-interventional design and to respect the current practice of healthcare professionals and caregivers, our study lacks the necessary evaluations of alternative dosage forms to compare their acceptability in the targeted age range. It must also be noted that the older population studied here consisted exclusively of patients in institutions with a distribution of treatment per unit dose by the internal pharmacy. Thus, the adequacy of the primary and secondary packaging has not been assessed for these patients; instruction leaflets were not read by patients as the medicinal products were prepared by healthcare professionals. In this controlled environment, some potential misuses which could have occurred in the home may not have been captured. It is important to note that acceptability profiles need to be considered with regards to the studied population, in a contextual environment. As such, these results cannot be directly extrapolated to the older home dwelling population, specific observations related to these patients is ongoing but the recruitement is notably slower.

In light of two 2017 reviews of acceptability testing in the paediatric and geriatric populations [18, 19], to the best of our knowledge this is the first study exploring the acceptability of various formulations of an API in real life conditions for both of these age groups. A contemporary review dedicated to the methodology used to assess acceptability of oral paediatric medicines [7] further corroborates this. According to Drumond, only “10 studies had used well-defined protocols and observational endpoints to investigate patient appropriateness”. Patient reported outcome tools (PRO), which are not recommended for use with children under 6 and questionable before 12 years of age [20], were used in many of the paediatric acceptability studies collected in these reviews. Alternatively, proxy questionnaires, which are not accepted by the Food and Drug Administration (FDA) [21], were used in other cited studies. In addition, those studies designed with a sole hedonic criterion were susceptible to generating misinterpretations. For example, in the entire population of older inpatients supporting the acceptability reference framework (n = 1288), positive patient reactions were overrepresented (55%) in only one class of treatments, ATC N02AA or natural opium alkaloids (n = 66). After interviewing the nurses involved, we understood that this positive reaction was generated upon announcing their arrival with the patient’s opioid, prior treatment administration. Therefore, the hedonic criteria did not measure the acceptability of the treatment with regards to the formulation, but rather the acceptability of the anticipated effect of the treatment. We also identified in the paediatric population that paracetamol 2.4% oral suspension was the sole medicinal product with a majority of positive reactions. This is certainly related to this population’s affinity for the strawberry aroma of this dosage form. As this could lead to unintended administrations, the health authorities prefer neutral reactions. By collecting many different variables for an analysis without weighting, this multivariate approach is able to simultaneously capture the range of factors comprised in the concept of acceptability. Three-dimensional mapping of these results places their barycentre position within a cluster profile that draws upon a reference framework, whereas in the literature acceptability is frequently limited to sequential comparisons of each specific criterion [18]. While the EMA guideline on pharmaceutical development of medicines for paediatric use states that "adequate patient acceptability is not to be understood as 100% acceptability of a medicine" [4], an 80% threshold appears to have been generally employed to date [7]. The use of aggregate scoring of acceptability criteria presented in this study better facilitates decisions regarding each medicinal product. If we take into consideration this study, the percentage of multivariate assessments per medicinal product falling within the “positively accepted” cluster failed to meet this 80% threshold in three of the five cases. As the barycentre positions and the confidence ellipses of all five of these dosage formulas belong to the cluster “positively accepted”, we consider that these multivariate analyses circumvent the need to assign an artificial threshold for acceptability as would be required when considering unidimensional criteria.

Paracetamol, with 124 marketed medicinal products in France, accentuates these challenges. Healthcare professionals must match the optimal set of characteristics to select a dosage form best adapted to individual patients of different ages, and each with a different set of underlying conditions. With this study, we set out to draw the attention of both clinicians and medical institutions to certain factors of particular interest. At the institutional level, we identified more than 16 paracetamol medicinal products referenced on hospital prescription lists, some of which were poorly adapted for the population targeted by the concerned facility. As discussed above, powder for oral solution was often administered to patients from the older inpatient population with a swallowing disorder, whereas our results indicate that it may not be the most appropriate dosage form. In a pragmatic approach to better understand the inclusion of such dosage forms in hospital dispensaries, working groups including all the actors of the hospital (i.e., nurses, physicians, pharmacists, administration and patient representatives) must be consulted. Observational studies might then be conducted to verify if any related or alternative dosage forms might be more accepted in the targeted population, and confirm their presence on the prescription list. Those dosage forms identified to cover the unmet need of a specific subpopulation, could subsequently be integrated into the institution’s standard practice through internal communication and training. A discrepancy in the use of administration devices was also uncovered by our study. For hygienic reason, additional oral administration syringes were purchased separately: in one hospital their volume was inferior to the original device, covering patient weighing from 3 kg to 8 kg instead of 3 kg to 13 kg. Unfortunately, 43% (n = 32/74) of the doses administered in this hospital were prescribed for patients >8 kg. The selection of this device imposed time-consuming additional handlings on the nursing staff, and more critically brought in a new risk factor for dosage error. We were not able to investigate the purchasing process of this syringe, but prior consultation of a multidisciplinary group would have improved the final process of oral suspension treatment administration in this setting.

A number of observations made in this non-interventional study call for further inquiry. Preliminary observations here revealed that ODT tended to be more suitable than the powder for oral solution in patients with swallowing disorders, a common complaint in older patients [22, 23]. In spite of a longer administration time, this dosage form was mainly related to positively connoted categories, but more evaluations in these patients with swallowing disorders are required. Ideally, this treatment option would spare individuals with dysphagia from the administration of powder for oral liquid solution. As some ODT contain sodium, however, potential cardiovascular risk contraindications among the older population must be considered prior to selecting which paracetamol ODT to stock. In parallel, two-thirds of the older patients treated with powder for oral solution had no swallowing disorder, we would be interested in verifying the acceptability the capsule dosage form in this population. Based on French public prices this would reduce these patients’ paracetamol budget by up to 25%. In the paediatric population, the place of the powder for oral solution might also be questioned: in this study, this dosage form was administered in children over nine that should be capable of swallowing capsules, and in children between three to five years eligible for treatment with the better accepted oral suspension. To address these questions, we have planned an interventional study to be conducted in hospitals, for both populations. Administration of a randomized adapted dosage form of paracetamol to the non-contraindicated population would be alternated over different weekdays using a cross-over methodology.

## Conclusions

Appropriate prescription of medicines extends beyond API and dosage levels. Although any number of dosage forms of an API may be accepted in the general population, additional care must be taken to integrate individual patient characteristics when prescribing for vulnerable populations. Selecting an appropriate adapted dosage form permits healthcare professionals to improve acceptability. In the case of paracetamol, institutions could be aided to reduce the number of medicinal products stocked in their dispensaries while retaining the necessary alternative dosage forms for vulnerable subpopulations. Further well-designed investigations will be vital to facilitate these processes to better serve these patients.

## Supporting information

S1 Table Demographic characteristics of the patients from the older population. (DOCX)

S2 Table Characteristics of the medicines assessed in the older population. (DOCX)

S3 Table Demographic characteristics of the patients from the paediatric population. (DOCX)

S4 Table Characteristics of the medicines assessed in the paediatric population. (DOCX)
