# Supplementary material for: Dosage form suitability in vulnerable populations: A focus on paracetamol acceptability from infants to centenarians
Source: PLoS One. 2019 Aug 20;14(8):e0221261. doi: 10.1371/journal.pone.0221261 (PMC6701828; doi:10.1371/journal.pone.0221261)
Supplement: S1 Table — (DOCX) [file pone.0221261.s001.docx]

**S1 Table. Demographic characteristics of the patients from the paediatric population**

| **Patients (n=1016)** | | | |
| --- | --- | --- | --- |
| **Characteristics** | | n | (%) |
| **Gender** | Girl | 475 | (48) |
|  | Boy | 522 | (52) |
|  | *md: 19* | | |
| **Age (years)** | [0, 2] | 483 | (48) |
|  | [3, 5] | 230 | (23) |
|  | [6, 8] | 133 | (13) |
|  | [9, 11] | 92 | (9) |
|  | [12, 14] | 45 | (5) |
|  | [15, 17] | 18 | (2) |
|  | *md: 15* | | |
| **Place** | Hospital | 284 | (28) |
|  | Ambulatory | 732 | (72) |
| *md: missing data* | | | |
