# Supplementary material for: Dosage form suitability in vulnerable populations: A focus on paracetamol acceptability from infants to centenarians
Source: PLoS One. 2019 Aug 20;14(8):e0221261. doi: 10.1371/journal.pone.0221261 (PMC6701828; doi:10.1371/journal.pone.0221261)
Supplement: S2 Table — (DOCX) [file pone.0221261.s002.docx]

**S2 Table. Characteristics of the medicines assessed in the paediatric population**

| **Medicines (n=283)** | | | |
| --- | --- | --- | --- |
| **Characteristics** | | n | (%) |
| **Formulations** | Powder for oral suspension | 55 | (19) |
|  | Oral solution | 21 | (7) |
|  | Oral suspension | 18 | (6) |
|  | Syrup | 16 | (6) |
|  | Coated tablet | 16 | (6) |
|  | *Other (2%<n<5%): drops for oral solution, powder for oral solution, tablet, orally disintegrating tablet, solution for inhalation, capsule, suspension for inhalation, ocular solution, granules for oral suspension, divisible tablet.* |  |  |
|  | *Other (n≤2%): oral lyophilisat, suppository, solution for injection, gastro resistant capsule, oral powder, oral gel, granules for oral solution, granules, divisible suppository, divisible effervescent tablet, coated divisible tablet, chewable tablet, suspension for injection, solution for spray, powder for solution for injection, powder for inhalation, gastro resistant tablet, effervescent tablet, dispersible tablet, suspension for spray, solution for application, rectal solution, prolonged release capsule, ointment, ocular ointment, nasal solution, granules sustained release, gastro resistant granules for oral suspension, emplatre, ear solution, divisible tablet for oral suspension, divisible dispersible tablet, dispersible or chewable tablet.* |  |  |
| **Anatomic therapeutic subgroups**  **(ATC level 2)** | Antibacterials for systemic use | 69 | (25) |
|  | Analgesics | 22 | (8) |
|  | Drugs for obstructive airway diseases | 21 | (8) |
|  | Corticosteroids for systemic use | 18 | (5) |
|  | Cough and cold preparations | 14 | (6) |
|  | Antihistamines for systemic use | 14 | (5) |
|  | *Other (2%<n<5%): drugs for acid related disorders, drugs for functional gastrointestinal disorders, anti-inflammatory and antirheumatic products, ophthalmologicals, psycholeptics, antiepileptics, antidiarrheals intestinal anti inflammatory anti infective agents, antianemic preparations.* |  |  |
|  | *Other (n≤2%): vitamins, drugs for constipation, mineral supplements, vaccines, all other therapeutic products, psychoanaleptics, antiprotozoals, antimycobacterials, stomatological preparations, nasal preparations, digestives including enzymes, antineoplasiques, antimycotics for systemic use, antiemetics and antinauseants, pituitary and hypothalamic hormones and analogues, otologicals, other alimentary tract and metabolism products, lipid modifying agents, immunosuppressants, cardiac therapy, calcium channel blockers, blood substitutes and perfusion solutions, beta blocking agents, antivirals for systemic use, antiseptics and disinfectants, antibiotics and chemotherapeutics for dermatological use, anthelmintics, anesthesics, allergens.* |  |  |
