# Supplementary material for: Dosage form suitability in vulnerable populations: A focus on paracetamol acceptability from infants to centenarians
Source: PLoS One. 2019 Aug 20;14(8):e0221261. doi: 10.1371/journal.pone.0221261 (PMC6701828; doi:10.1371/journal.pone.0221261)
Supplement: S3 Table — (DOCX) [file pone.0221261.s003.docx]

**S3 Table. Demographic characteristics of the patients from the older population**

| **Patients (n=1288)** | | | |
| --- | --- | --- | --- |
| **Characteristics** | | n | (%) |
| **Gender** | Women | 879 | (69) |
|  | Men | 393 | (31) |
|  | *md^a^: 16* | | |
| **Age (years)**  *Mean: 86.4 sd(7.2)* | [65, 75) | 77 | (6) |
|  | [75, 85) | 409 | (32) |
|  | [85, 95) | 653 | (51) |
|  | [95, +∞) | 143 | (11) |
|  | *md: 6* | | |
| **Place** | Hospital | 1044 | (81) |
|  | Nursing home | 244 | (19) |
| **Disorders** | Swallowing disorder | 222 | (17) |
|  | Muscular or rheumatologic disorders of the upper limbs | 293 | (23) |
|  | Memory disorder | 765 | (60) |
| **IADL Scale^b^** | 4 *(high function, independent)* | 126 | (12) |
|  | 3 | 119 | (11) |
|  | 2 | 153 | (15) |
|  | 1 | 323 | (31) |
|  | 0 *(low function, dependent)* | 321 | (31) |
|  | *md: 246* | | |
| **Number of prescribed medicines per day** | [1, 5) | 87 | (6) |
|  | [5, 10) | 521 | (42) |
|  | [10, +∞) | 647 | (52) |
|  | *md: 33* | | |
| *^a^ md: missing data*  *^b^ Overall score calculated as a sum of the four items. For each item, a score of 1 defined an autonomous patient while a score of 0 a dependent one.* | | | |
