# Supplementary material for: Dosage form suitability in vulnerable populations: A focus on paracetamol acceptability from infants to centenarians
Source: PLoS One. 2019 Aug 20;14(8):e0221261. doi: 10.1371/journal.pone.0221261 (PMC6701828; doi:10.1371/journal.pone.0221261)
Supplement: S4 Table — (DOCX) [file pone.0221261.s004.docx]

**S4 Table. Characteristics of the medicines assessed in the older population**

| **Medicines (n=315)** | | | |
| --- | --- | --- | --- |
| **Characteristics** | | n | (%) |
| **Formulations** | Capsule | 39 | (12) |
|  | Divisible tablet | 39 | (12) |
|  | Coated tablet | 37 | (12) |
|  | Divisible coated tablet | 36 | (11) |
|  | Tablet | 34 | (11) |
|  | *Other (2%<n<5%): oral solution, powder for oral solution, capsule sustained release,orally disintegrating tablet..* |  |  |
|  | *Other (n≤2%): oral solution in drops, solution for injection, coated tablet sustained release, tablet sustained release, effervescent tablet, dispersible tablet, oral suspension, powder for oral suspension, patch, enrobed tablet, collyre, syrup, capsule gastro-resistant, tablet gastro-resistant, suspension for inhalation, solution for inhalation, powder for inhalation, granule sustained release, oral gel, divisible coated tablet sustained release, gastro-resistant enrobed tablet, lozenge.* |  |  |
| **Anatomic therapeutic subgroups**  **(ATC level 2)** | Analgesics | 43 | (14) |
|  | Psycholeptics | 40 | (13) |
|  | Psychoanaleptics | 37 | (12) |
|  | Antiepileptics | 25 | (8) |
|  | Drugs used in diabetes | 20 | (6) |
|  | *Other (2%<n<5%): antithrombotic agents , agents acting on the renin–angiotensin system, beta blocking agents, calcium channel blockers, anti-parkinson drugs, drugs for constipation.* |  |  |
|  | *Other (n≤2%):* *mineral supplements, antianemic preparations, thyroid therapy , ophthalmologicals, drugs for acid related disorders, antibacterials for systemic use, lipid modifying agents, other nervous system drugs, drugs for obstructive airway diseases, corticosteroids for systemic use, diuretics, antihypertensives, urologicals, medicaments en cardiologie, antivirals for systemic use, vasoprotectives, tous autres medicaments, muscle relaxants, endocrine therapy, drugs for functional gastrointestinal disorders, antiprotozoals, antimycotics for systemic use, antimycobacterials, anti inflammatory and antirheumatic products, antihemorrhagics, antidiarrheals intestinal anti inflammatory anti infective agents.* |  |  |
